# Atypically Protracted Course of Liver Involvement in Kawasaki Disease. Case Report and Literature Review

**DOI:** 10.3390/pediatric13030044

**Published:** 2021-07-01

**Authors:** Pamela Paglia, Lucia Nazzaro, Anna Giulia Elena De Anseris, Milena Lettieri, Rossella Colantuono, Maria Chiara Rocco, Maria Anna Siano, Nicola Biffaro, Pietro VAJRO

**Affiliations:** 1 Chair of Pediatrics and Residency Program of Pediatrics, Department of Medicine, Surgery and Dentistry “Scuola Medica Salernitana”, University of Salerno, 84081 Baronissi, Italy; pagliapamela90@gmail.com (P.P.); lettieri.milena@libero.it (M.L.); rossella.colantuono31@gmail.com (R.C.); mariachiara@live.it (M.C.R.); mariaannasiano@gmail.com (M.A.S.); nicola.biffaro@libero.it (N.B.); 2 Pediatrics Units of San Giovanni di Dio e Ruggi d’Aragona University Hospital, 84131 Salerno, Italy; nazzaroluci@gmail.com (L.N.); annagiulia.deanseris@gmail.com (A.G.E.D.A.); 3 Department of Translational Medical Sciences, Section of Pediatrics, “Federico II” University, 80131 Naples, Italy

**Keywords:** Kawasaki disease, persistent hypertransaminasemia, case report, literature review

## Abstract

Hypertransaminasemia in patients with Kawasaki disease (KD) is reported to be transient. Here, we describe a child with an atypically protracted course of liver tests abnormalities and review the inherent literature. The patient was hospitalized at age 7-months for isolated hypertransaminasemia detected during a classical KD diagnosed 3 months before, and persistent since then. KD clinical evolution had been favorable, with rapid response to acetylsalicylic acid and intravenous immunoglobulins. Liver enzymes however remained persistently elevated with a fluctuating pattern (ALT > AST levels; peak of AST 186 IU/L and ALT 240 IU/L). During follow-up, the main causes of liver dysfunction had to be excluded through appropriate and extensive laboratory investigations. Transaminases values become steadily normal only 7 months after the acute presentation of KD. Conclusions: Our report shows that an atypically protracted courses of KD-related hypertransaminasemia above the previously reported temporal limits should be taken into account during the stepwise diagnostic approach to the patient’s liver dysfunction. Insidious acetylsalycilic acid-hepatotoxicity warrants consideration in the differential diagnosis.

## 1. Introduction

Kawasaki disease (KD) is a systemic inflammation in all the medium-sized arteries and in multiple organs and tissues during the acute febrile phase. Common gastrointestinal findings include diarrhea, vomiting, abdominal pain, hepatitis, and gallbladder hydrops while pancreatitis and jaundice are less common. Elevations in serum aminotransferases and/or gammaglutamyl-transpeptidase (GGT) documented in about one third/one half of patients are mild to moderate [1,2,3,4,5] and are described to be transient as they improve rapidly with the resolution of acute symptoms [6]. Here, we describe the case of a KD patient with an atypically protracted course of hypertransaminasemia and focus on recovery time of abnormal liver function tests in this condition.

## 2. Case Report

L.S., male, was born at term after an uneventful pregnancy and vaginal delivery. He was breastfed. At age 4 months, he was hospitalized because of a 4-day-long fever resistant to paracetamol/ibuprofen. At entry, classical KD diagnosis was made (five clinical criteria in addition to fever) and he received high-dose acetylsalicylic acid (ASA) until 48 h after persistent defervescence. (i.e., 80 mg/kg/day × 5 days), subsequently switched to low-dose (3 mg/kg/day) for a total duration of 8 weeks after the onset of disease. There was no recent history of other medications or viral infection. The clinical evolution of KD was favorable, with rapid response to intravenous immunoglobulin (IVIG) and no coronary involvement. Because of persistent isolated hypertransaminasemia with normality of the remaining liver and muscular tests, at the 9th week of disease, the patient was addressed to our pediatric hepatology unit. At entry, he was aged 7 months, was anicteric, and had a liver margin palpable 2.5 cm below the costal arch, without clinical signs of severe liver disease. Spleen was not enlarged. Mental status was and remained normal overtime. After 2 further weeks of isolated liver enzymes abnormalities persistence, the main causes of KD-related and unrelated hypertransaminasemia due to infectious [including blood cultures, polymerase chain reaction studies for adenovirus, cytomegalovirus, and Epstein-Barr virus, hepatitis A virus (HAV), hepatitis B virus (HBV), hepatitis C virus (HCV) screening test], autoimmune [antinuclear antibodies (ANA), anti-smooth muscle antibodies (ASMA), anti-mitochondrial antibodies (AMA), anti-liver kidney microsome type 1 (LKM1), anti-neutrophil cytoplasmic antibodies (ANCA), serum proteins and immunoglobulins], metabolic/genetic (including serum alpha1 antitrypsin, ceruloplasmin, glucose, ammonium and copper, and sweat test), nutritional/intestinal (serologic test for celiac disease), toxic, muscular (creatine phosphokinase, CPK) conditions were all excluded through appropriate laboratory investigations. The abdominal ultrasounds were negative for gallbladder hydrops, steatosis, and other signs except for a mild hepatomegaly.

Aminotransferase values strictly monitored during the first 3 months and nearly every 1–2 months thereafter persisted elevated with a slightly fluctuating pattern and a quite continue tendency of ALT levels > AST levels. They become steadily normal only 7 months after the diagnosis of KD.

## 3. Discussion and Conclusions

Liver involvement in KD is commonly subclinical and self-limiting. In a large retrospective study of 240 patients with KD patients, approximately one third had hypertransaminasemia with only mildly elevated aminotransferases values, i.e., less than twice the upper limit of normal. Only a few presented with a picture of clinical hepatitis with jaundice and aminotransferase elevations of more than 10-fold [2].

The natural recovery time of liver function tests in KD has been rarely studied. As summarized in Table 1, it has been reported only in one series where it appears to be 7 days (median), ranging from 2 days to 99 days [6], and in one case report where aminotransferases recovered after 4 months from onset [7]. Another series describes only an early tendency to recovery in the first/second week of illness [8].

The much longer duration of liver abnormalities with a slow decrease of aminotransferase values at considerable distance from initial diagnosis of KD observed in our patient suggests that hypertransaminasemia may occasionally be more protracted than previously thought.

Hepatobiliary enzymes elevations in KD may reflect a number of underlying manifestations ranging from asymptomatic increase in liver enzymes to severe cholestatic hepatitis, acute acalculous cholecystitis, or hydrops of the gallbladder [10]. Though hepatic disease per se is not a significant cause of morbidity or mortality in KD patients, monitoring of abnormal liver function is important because it may mirror the severity of ongoing systemic inflammation, which has been reported to affect in some manner the efficacy of IVIG treatment. Early recognition of liver function tests abnormalities may help to intercept an IVIG resistant disease, a condition which tends to be more prevalent in those who develop coronary artery abnormalities [11]. High AST levels and abnormal ultrasonographic biliary findings moreover have been considered risk factors for coronary artery abnormality and/or recurrent KD if they are present during the first episode [7,10,12].

KD is a childhood systemic vasculitis which still remains without a well-defined etiology. The epidemiology and clinical presentation suggest a possible underlying viral/bacterial infection or an abnormal immunologic response to an infection in genetically predisposed children; autoimmune disease is a possibility as well [13,14]. After exhaustive investigation, we were not able to find any of the above-mentioned causative triggers of KD in our patient and therefore we are not aware if the agent triggering KD was also hepatotropic and responsible for both the vasculitis and this atypically prolonged liver involvement. Alternatively, the liver involvement was due to a concomitantly associated but unrelated liver insult deriving by another cause. Considering that, the prolonged course of the liver test abnormalities of our patient required to exclude the entire series of the most frequent liver infections, autoimmune, metabolic, or toxic causes [15].

**Figure 1:**
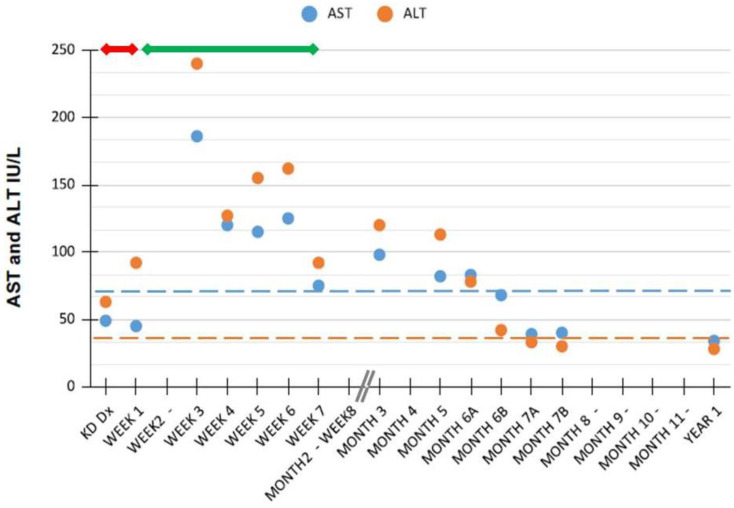
Aminotransferases values after diagnosis of Kawasaki disease in our patient. Horizontal dashed lines indicate ALT and AST upper normal values for age [16]; the horizontal continuous red and green lines indicate treatment duration with acetylsalicylic acid at high and low doses, respectively. Abbreviations: ALT, alanine aminotransferase; AST, aspartate aminotransferase; Dx, diagnosis; KD, Kawasaki disease.

Pathologic findings of the liver described in KD patients with hepatic presentation or in autopsies encompass a variety of sinusoids and portal areas lesions, and/or swelling of Kupffer cells, fatty degeneration, and severe congestion. Regarding the latter, therefore, postulated mechanisms underlying liver function tests abnormalities in these patients suggest to include also congestive heart failure secondary to myocarditis, drug induced liver injury (so called DILI), and/or a combination of the above [2]. As shown in Figure 1, in our patient, the temporal relationship between ASA treatment and the highest aminotransferases values might indeed imply also a DILI mechanism.

Hepatotoxicity of ASA through mitochondrial dysfunction, higher hepatic fatty acid supply, and microvesicular steatosis could explain at least in part the dose dependent and usually transient and asymptomatic hepatic dysfunction seen in KD [17,18]. As liver failure is generally observed mainly when ASA is used at medium-high dosage, aminotransferase level monitoring is mandatorily recommended to either reduce dosage or stop ASA treatment. In connection with chickenpox and flu, the risky possibility of Reye’s syndrome should be seriously considered although, at low dosage of ASA, the occurrence of this syndrome is reported to be rare [19,20]. In our case, the modest transaminase peak observed during aspirin treatment along with the other clinical and laboratory data make improbable a Reye’s syndrome like related hepatotoxicity in addition to the usual KD-related transient hypertransaminasemia. Still, an atypically prolonged idiosyncratic ASA hepatotoxicity remains an open question.

In spite of these premises, it is worth of note that ASA frequency of use and doses (ranging 10–30 mg/kg/day) are reported having been not significantly different in 381 KD patients (mean age 25.3 months) divided in three groups allocated into children with ALT level ≥40 IU/L on admission, ≥40 IU/L at some point after admission, or ALT levels consistently <40 IU/L throughout hospitalization [9]. Still, these authors considered an arbitrary upper normal value of transaminases, which is not appropriate during the first year of life. (Table 1)

In conclusion, our report shows the possibility of facing an atypically protracted course of KD-related hypertransaminasemia above the hitherto reported temporal limits. Due to the likely uncommonness of the event here described, those infectious/immune causes which could have acutely triggered both conditions still need to be investigated in first instance. Should hypertransaminasemia persist, pending specific markers of KD-related liver dysfunction, a stepwise approach with careful evaluation of the cost–benefit ratio in the use of additional diagnostic resources is anyway warranted to search for an underlying KD-unrelated persistent/chronic hepatopathy [15]. Because ASA doses recommended by the American Academy of Pediatrics and the American Heart Association to attain an anti-inflammatory effect during the acute phase of KD are fairly high, reaching up to 80–100 mg/kg per day, high dose-ASA-related DILI risk remains a possible insidious confounder in KD. The question of whether the benefits of high dose ASA warrants its continued use in KD however is not likely to get an answer presently since all prospective studies that have demonstrated the effectiveness of IVIG in treating KD also employed moderate to high doses of aspirin [21].

## Figures and Tables

**Table 1:** Natural recovery time of liver enzymes abnormalities in Kawasaki disease.

Author	Case Report/Case Series	Transaminases Values at Diagnosis		Recovery Time from Onset		Treatment with Acetylsalicylic Acid
	N; Gender; Age	AST IU/L	ALT IU/L	AST IU/L	ALT IU/L	
**Jang 2016** [6]	63 patients 2.3 ± 1.7 years	>50	>50	7 days (median); range 2–99 days		NR
**Keeling 2016** [7]	1 F 12 years	17	42	4 months	4 months	500 mg three times a day during 10-days hospitalization and continued after discharge
**Tomita 2019** [8]	199/381 patients (52.2%) 97 F–102 M 27.2 ± 22.5 months	NR	(I) ALT ≥ 40 *on admission*	AST, peaked on days 1–3 and tended to exhibit recovery on the 4th day of illness	ALT began to normalize on the 6th day of illness	37/199 patients (18.6%) received aspirin at doses of 10–30 mg/kg/day
	52/381patients (13.6%) 16 F–36 M 20.9 ± 25.6 months	NR	(II) ALT ≥ 40 at some point *after admission*	AST, peaked on 11th day and tended to exhibit recovery on 15th–17th day of illness	ALT decreased on 15th–17th days of illness	10/52 patients (19.2%) received aspirin at doses of 10–30 mg/kg/day
**Rohani 2020** [9]	1 M 16 months	351	40	Persistent elevation after 9 months (Macro-AST)	2 weeks	35 mg/kg/day divided q6h for 48 h from diagnosis and reduced after 36 h to 3 mg/kg once daily until 6 wk after illness onset
**Present** **Case**	1M 4 months	50 (at Dx)	60 (at Dx)	AST peaked at week 3 (186 IU/L); tended to normalize at 6th-7th month	ALT peaked at week 3 (240 IU/L); tended to normalize at 6th–7th month	80 mg/kg/day × 5 days then 3 mg/kg/day × total 8 weeks

Abbreviations: ALT, alanine amino transaminase; AST, aspartate amino transaminase; Dx, Diagnosis; F, female; M, male; NR, not reported.

## Data Availability

No additional data sets are associated with this paper.
